# Human growth is associated with distinct patterns of gene expression in evolutionarily conserved networks

**DOI:** 10.1186/1471-2164-14-547

**Published:** 2013-08-13

**Authors:** Adam Stevens, Daniel Hanson, Andrew Whatmore, Benoit Destenaves, Pierre Chatelain, Peter Clayton

**Affiliations:** 1 Manchester Academic Health Sciences Centre, Faculty of Medical and Human Sciences, Royal Manchester Children’s Hospital and the Institute of Human Development, University of Manchester, Manchester, UK; 2 Merck Serono S.A, Geneva, Switzerland; 3 Department Pediatrie, Hôpital Mère-Enfant, Université Claude Bernard, Lyon, France

**Keywords:** Development, Evolution, Gene expression, Growth, Network analysis, Pediatrics

## Abstract

**Background:**

A co-ordinated tissue-independent gene expression profile associated with growth is present in rodent models and this is hypothesised to extend to all mammals. Growth in humans has similarities to other mammals but the return to active long bone growth in the pubertal growth spurt is a distinctly human growth event. The aim of this study was to describe gene expression and biological pathways associated with stages of growth in children and to assess tissue-independent expression patterns in relation to human growth.

**Results:**

We conducted gene expression analysis on a library of datasets from normal children with age annotation, collated from the NCBI Gene Expression Omnibus (GEO) and EBI Arrayexpress databases. A primary data set was generated using cells of lymphoid origin from normal children; the expression of 688 genes (ANOVA false discovery rate modified p-value, q < 0.1) was associated with age, and subsets of these genes formed clusters that correlated with the phases of growth – infancy, childhood, puberty and final height. Network analysis on these clusters identified evolutionarily conserved growth pathways (NOTCH, VEGF, TGFB, WNT and glucocorticoid receptor – Hyper-geometric test, q < 0.05). The greatest degree of network ‘connectivity’ and hence functional significance was present in infancy (Wilcoxon test, p < 0.05), which then decreased through to adulthood. These observations were confirmed in a separate validation data set from lymphoid tissue. Similar biological pathways were observed to be associated with development-related gene expression in other tissues (conjunctival epithelia, temporal lobe brain tissue and bone marrow) suggesting the existence of a tissue-independent genetic program for human growth and maturation.

**Conclusions:**

Similar evolutionarily conserved pathways have been associated with gene expression and child growth in multiple tissues. These expression profiles associate with the developmental phases of growth including the return to active long bone growth in puberty, a distinctly human event. These observations also have direct medical relevance to pathological changes that induce disease in children. Taking into account development-dependent gene expression profiles for normal children will be key to the appropriate selection of genes and pathways as potential biomarkers of disease or as drug targets.

## Background

Height growth in humans is distinctly different from growth in other mammals and is characterised in infancy by rapid but decelerating growth, a prolonged childhood phase of slow growth and a pubertal growth “spurt” before final height is reached – termed the Infancy/Childhood/Puberty (ICP) growth model [1]. Growth in rodents along with most other animals follows a very different path with maximum growth rate occurring in the early postnatal period, rapid growth to sexual maturity and much slower growth thereafter [2,3].

Variation in the timing or rate of developmental events is recognised as a mechanism for evolutionary change. This phenomenon can be observed in the origin of the elongated childhood (juvenile) growth phase in humans, which can also be seen in social mammals and is associated with the learning of complex behaviours required for survival [2,4]. In puberty primates show some associated body-weight gains but no evidence of a return to the long-bone elongation seen in humans, suggesting that the growth spurt associated with human puberty is specific to *Homo sapiens* as supported by both comparative auxological studies [2] and the fact that human height growth can only be defined by complex mathematical modelling in comparison to simpler models for other primates [2,5].

As yet no study has examined the changes in gene expression that accompany development in children and correlated them with biological pathways at different ages and stages of development. Some insight into the genetic program that accompanies growth in mammals has been gained from the study of rodent models where tissue-independent gene expression has been shown in a range of cell types [6]. This work provided mechanistic insight into the existence of a multi-organ genetic program to suppress growth [6,7] which was associated with human imprinted genes some of which are involved with the etiology of rhabdomyosarcoma [8]. It is likely that a genetic program could exist to underpin human height growth, but it would need to relate not only to growth deceleration, but also acceleration as seen in puberty.

The use of lymphoid tissue as a tool to study human growth response has been established [9,10]. Gene expression profiles between multiple types of human tissue is marked by their similarity (>30%) with only a limited fraction of gene expression being truly tissue specific (<15%) [11-13]. It is recognised that the control of gene expression between tissues is highly independent in relation to specific functions [14] but redundancy does exist in relation to “maintenance” (“house-keeping”) functions, including growth related genes [13,14].

In this analysis, we have used transcriptomic data from cells of lymphoid origin to describe gene expression clusters that correlate with the phase of childhood growth. We have then used network analysis to examine gene expression clusters in relation to a map of all known human protein and genetic interactions (the Interactome). To understand the biological pathways associated with human development/height growth and age, we have used derived sets of the most highly communicating proteins, i.e. those with high “connectivity” [15] and therefore the greatest functional relevance [16]. Finally, we have compared development-related gene expression in a number of different human tissues to highlight candidate pathways associated with a multi-organ genetic program of growth.

## Results

### Development-related gene expression correlates with the phases of human growth

**Figure 1:**
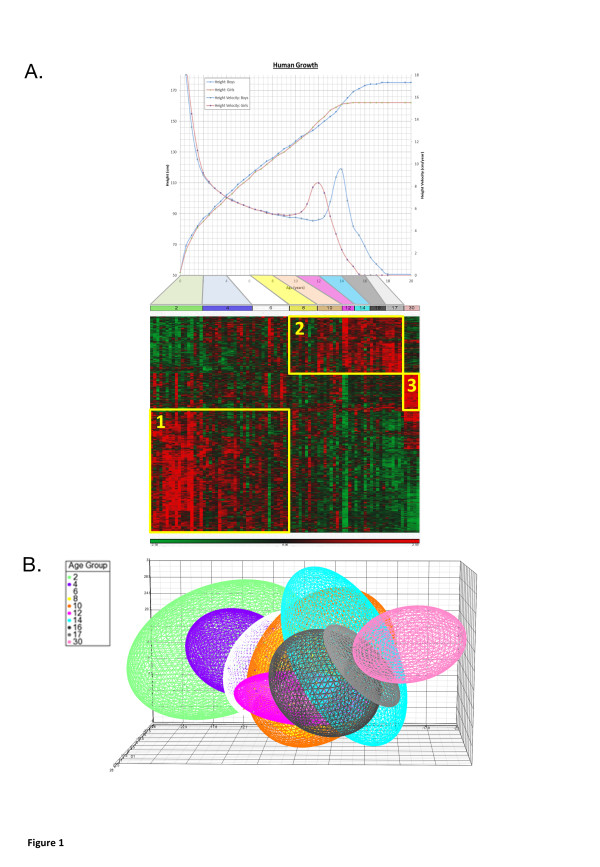
**Differential age-associated gene expression. A**. Heat map of age-associated changes in gene expression in 87 individuals (0.2 to 29.3 years of age - average 7.7+/−7.0 yrs). 927 gene expression probes were significantly associated with age (ANOVA, false discovery rate (FDR) corrected p-value, q < 0.1, with both gender and study used as co-variates. Supervised hierarchical clustering using Kendell’s dissimilarity and Ward’s method identified three main clusters of gene expression probes: ≤6 years of age [infancy, early childhood group (408 probes)]; >6 to ≤17 years of age [late childhood, puberty group (252 probes)]; and >17 to <30 years of age [adulthood (267 probes)]. Gene expression is shown between +2.5 fold and −2.5 fold, red = increase in gene expression, green = decrease in gene expression. Human growth curve data from normal controls [18,19] shown in relation to the heat map, age groups coloured by upper limit of group bins (years). **B.** Multi-dimensional scaling (MDS) of the 927 development-related gene expression probes shows distinct development-related clustering; ellipsoids represent 2 standard deviations of normalised gene expression, colour coded by age. Axes represent proportion of variation as defined by MDS (%).

We conducted a gene expression analysis on a library of datasets from normal children with age annotation, collated from the NCBI Gene Expression Omnibus (GEO) and EBI Arrayexpress (Additional file 1: Table S1). These four data sets were then combined to form a study group of 87 individuals ranging from 0.2 to 29.3 years of age (yrs) (average 7.7+/−7.0 yrs) (Additional file 1: Table S1 & Additional file 2: Figure S1). There were 927 gene expression probes significantly associated with age representing 688 distinct genes (false discovery rate adjusted p-value, q < 0.1), 477/688 of these age-related genes were associated with growth ontology (p<3.4 × 10^-3^). The 688 age-related genes found formed three distinct clusters of higher gene expression: 1) ≤6 yrs [infancy, early childhood] (408 probes representing 276 genes); 2) >6 to ≤17 yrs [late childhood, puberty] (252 probes representing 207 genes) and 3) >17 yrs [adulthood, final height] (267 probes representing 205 genes) (Figure 1 & Additional file 1: Table S2). These gene clusters demonstrated age-related differences in associated gene ontology and canonical pathways (Additional file 2: Figures S2 & S3, Additional file 1: Table S3) and for predicted transcription factor (TF) recruitment to promoter elements in these genes (Additional file 2: Figure S4). Transcription factors included Early B Cell Factor 1 (EBF1) and PAX5 (Additional file 2: Figure S4) that have been associated with both the proliferation of B cells and also the regulation of bone development [17].

Of the genes associated with age, 175 had been previously identified in human genome wide association studies (GWAS) on a variety of different conditions [20]; including 11 genes associated with human height (p < 0.007), one of which was the top age-associated gene in our study *IGF2BP3* (q < 1.19 × 10^-18^) (Additional file 1: Table S4). These genes are therefore both associated with the phases of human growth as well as final adult height. In addition a further 9 genes were associated with diabetes (p < 0.006), including *BACH2* (q < 1.5 × 10^-10^), the second most significantly age-associated gene in this study.

Recently age-dependent changes have been observed in the methylation of human genes in peripheral blood mononuclear cells (PBMCs) [21]. Of the genes in our study identified as having age-dependent alterations in gene expression, 12% (80/688) also have age-associated methylation changes (p < 0.05) [21]. The age-related genes were also mapped onto mouse genes identified as having STAT5 bound upon activation by growth hormone (GH) to examine putative GH regulation [22]; 18% (124/688) were associated with STAT5 in this model (p < 0.001). It was also noted that of the five genes chosen as representative of multi-tissue age-regulated down-regulation and used to define the rodent common program of growth deceleration [23], *IGF2BP3, SOX4* and *MEST* were present in the 688 age-related genes (Additional file 1: Table S2) and *PEG3* and *IGF2* were not tested for in this study.

### Connectivity of genes corresponds with growth-phase related expression

**Figure 2:**
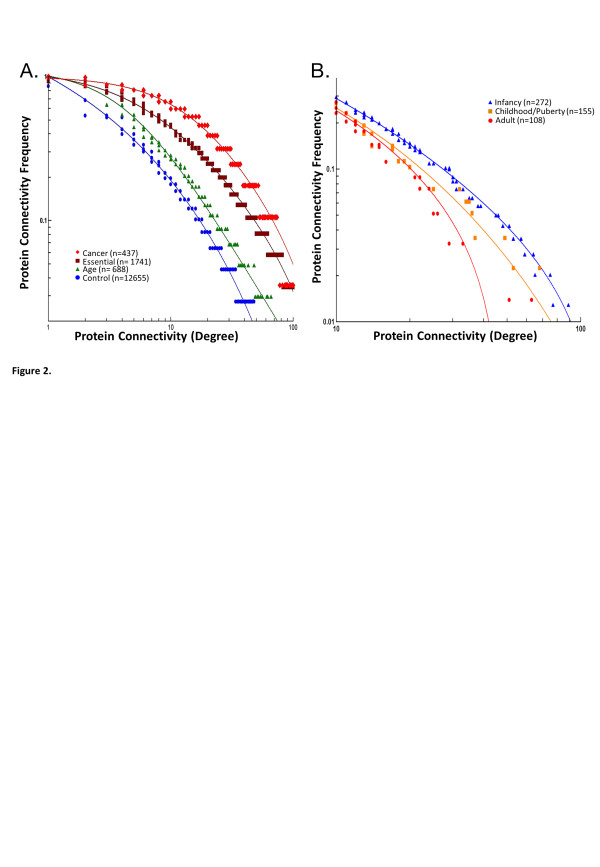
**Analysis of protein connectivity (degree) in the human interactome as a measure of protein function.** Protein connectivity was measured from a model of the human interactome (Biogrid build 3.1.87) and plotted against the frequency of proteins of specific degree; a right shift indicates an increase in connectivity and implies increased functional relevance [15,16]. **A)** Comparison of proteins with age related differences in expression [green marker n = 688] with genes associated with cancer (Cancer Gene Census database [25]) [red marker n = 437], “essential” genes derived from human orthologs of mouse lethal genes [26] [brown marker n = 1741] and control genes [16] [blue marker n = 12655]. All data sets are significantly different p < 0.0001, Wilcoxon test. **B)** Comparison of the connectivity within the human interactome proteins associated with gene expression changes during human growth; infancy, blue marker n = 272; Childhood/Puberty, orange marker n = 155; Adult/Final height, red marker n = 108. All data sets are significantly different p < 0.0001, Wilcoxon test.

The development-associated clusters of genes defined by expression analysis were used to generate interactome models based on all known protein:protein and protein:genetic interactions (Biogrid Homo sapiens 3.1.87) [24]. Examination of the development-associated clusters of genes within the human interactome demonstrated a significant increase in their ‘connectivity’ (protein degree score) compared to controls (p < 0.0005, Figure 2A). Interactome connectivity is a marker of biological function and associated with “essential” biological mechanisms [15,16]. The highest levels of protein connectivity were shown to occur during infancy and to reduce as final height is reached (Figure 2B).

### Minimal essential networks derived from growth phase-related gene expression are associated with evolutionarily conserved growth pathways

**Figure 3:**
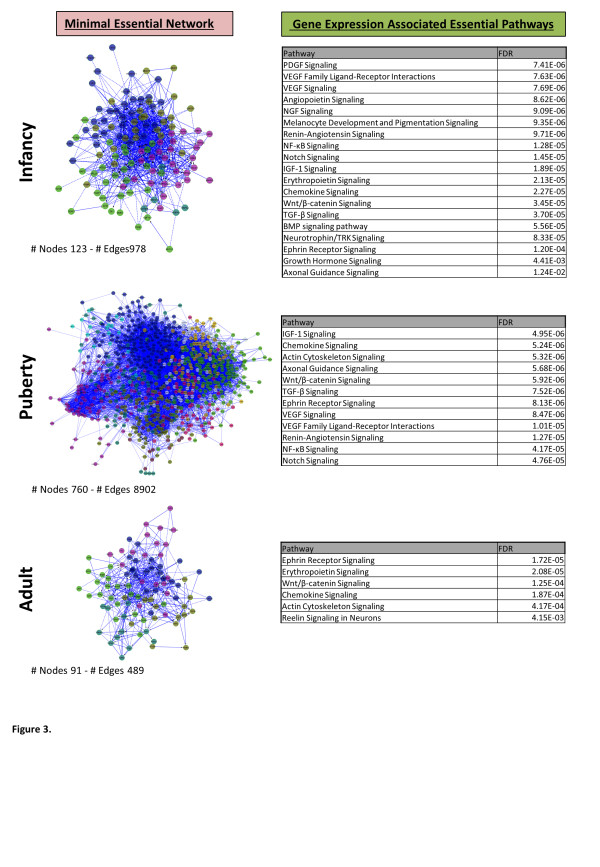
**Gene expression associated essential pathways correlated with phases of human growth.** The genes from the three development-related clusters; ≤6 yrs [Infancy, Early Childhood]; >6 to ≤17 yrs [Late Childhood, Puberty] and >17 yrs [Adult, Final Height] and were used to generate inferred networks from the human interactome using the Biogrid Cytoscape plugin; the top 10% of “hubs” and “bottlenecks” within the network were identified using the Cytohubba algorithm and used to generate minimal essential networks (shown). Pathway associations were assigned using the Reactome database [27-29]. Growth factor and organismal growth signalling pathway ontology defined from differential gene expression (Ingenuity Pathway Analysis) was correlated with the minimal essential networks and the resultant gene expression associated essential pathways tabulated (hypergeometric test, Benjamini-Hochberg false discovery rate correction [FDR]).

**Figure 4:**
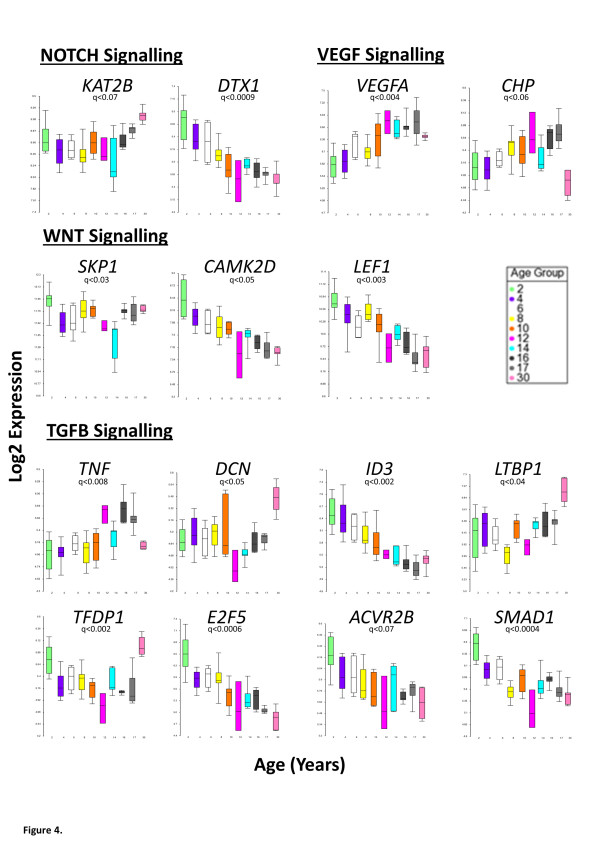
**Significant development-related changes in the NOTCH, VEGF, TGFB and WNT pathways as identified by the analysis of the essential properties of gene networks.** Exemplar genes from each of these pathways are presented (ANOVA, false discovery rate (FDR) corrected p-value, q < 0.1). Box and whisker plot (whiskers = top and bottom 10%) of gene expression as Log2 normalised values (y-axis). The x-axis is age-banded in years (upper limit of age group bin).

Minimal essential networks (MEN) consist of the top 10% of interactome proteins scored for connectivity and bottleneck network properties, and represent the most functionally related regions of interactome models [15,16] (Additional file 2: Figure S5). MEN were derived from the interactome models generated from the different gene expression clusters correlating with stage of child development (Figure 1). We used MEN gene expression changes to detect growth related evolutionarily conserved biological pathways (Additional file 2: Figure S5, Figure 3 & Additional file 1: Table S5) including NOTCH, VEGF, TGFB and WNT pathways in both the infancy/early childhood and late childhood/puberty clusters of gene expression. The infancy phase of human growth is characterised by a rapid deceleration and the gene expression identified in the infancy/early childhood associated cluster (Figure 1) demonstrated a similar deceleration of expression, for example *DTX1* in the NOTCH signalling pathway, *CAMK2D* and *LEF1* in the *WNT* signalling pathway and *ID3* and *E2F5* in the *TGFB* signalling pathway (ANOVA q < 0.1, Figure 4). The puberty phase of human growth is characterised by a return to growth and there were also distinct gene expression profiles in the late childhood/puberty associated cluster of gene expression (Figure 1) that showed a corresponding rate of change including *VEGFA* and *CHP* in the VEGF signalling pathway (positive correlation), *SKP1* in the WNT signalling pathway (negative correlation) and *TNF* (positive correlations), *DCN* and *TFDP1* (negative correlation) in the TGFB signalling pathway. In the adult cluster of gene expression (Figure 1) the genes showed unchanged gene expression throughout childhood with a final rapid increase in gene expression including *LTBP1* in the TGFB pathway and *KAT2B* in the NOTCH pathway (ANOVA q < 0.1, Figure 4).

### Gene expression of glucocorticoid receptor interacting proteins correlate with the phases of human growth

**Figure 5:**
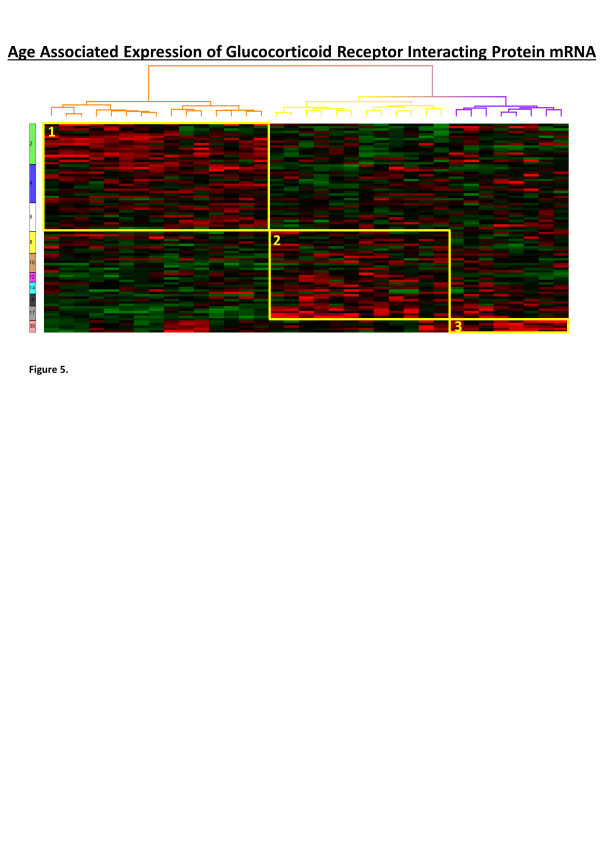
**Heat map of age-associated changes in the expression of genes for glucocorticoid receptor interacting proteins (n = 338) in 87 individuals (0.2 to 29.3 years of age - average 7.7+/−7.0 yrs); 35 gene expression probes were significantly associated with age (ANOVA, false discovery rate (FDR) corrected p-value, q < 0.2).** Supervised hierarchical clustering using Kendell’s dissimilarity and Ward’s method identified three main clusters of gene expression probes correlating with ≤6 years of age [infancy, early childhood group (15 probes)]; >6 to ≤17 years of age [late childhood, puberty group (12 probes)] and >17 to <30 years of age [adulthood (8 probes)]. Gene expression is shown between +2.5 fold and −2.5 fold, red = increase in gene expression, green = decrease in gene expression.

Analysis of human interactome associated canonical pathways demonstrated the key involvement of glucocorticoid receptor (GR) mediated transcription in all age groups. Glucocorticoid regulation has been shown to modulate function in NOTCH, VEGF, TGFB and WNT signalling [30]. The GR has well established interactions with the GH pathway, mainly mediated by regulation of IGF-I but also by cross-talk with STAT3 and STAT5 [31]. Also glucocorticoid excess in childhood, either as endogenous hypersecretion in Cushing’s syndrome or as exogenous therapy, has a marked growth inhibitory effect. To further investigate whether GR modulation was associated with age, we generated a list of current known direct interacting proteins of the GR (338 interactions defined from Ingenuity Knowledge Base) and examined how the expression of these genes changed with age (ANOVA, p < 0.05). Three distinct clusters of increased expression of GR interacting genes were observed correlating with the infancy, early childhood group (15 probes), the late childhood, puberty group (12 probes) and the adult group (8 probes) (Figure 5).

### A minimal essential network derived from tissue-independent development-related gene expression is associated with evolutionarily conserved growth pathways

The presence of a set of evolutionarily conserved growth pathways associated with the phases of human growth and development in lymphoid tissue was validated by replication in an independent set of PBMC gene expression data from age-grouped normal children (Additional file 1: Tables S1 & S6). In this data set (n = 53) no adult controls were available but infancy and late childhood/puberty clusters of development-related gene expression were defined including 549 genes (753 probe-sets, ANOVA, p < 0.01). Derived interactome models were associated with the same biological pathways identified using the main data set (Additional file 1: Table S6); 16/19 identical biological pathways in infancy & 9/12 identical biological pathways in late childhood/puberty (as identified by Ingenuity Pathway Analysis [IPA]) (Additional file 1: Tables S5).

**Figure 6:**
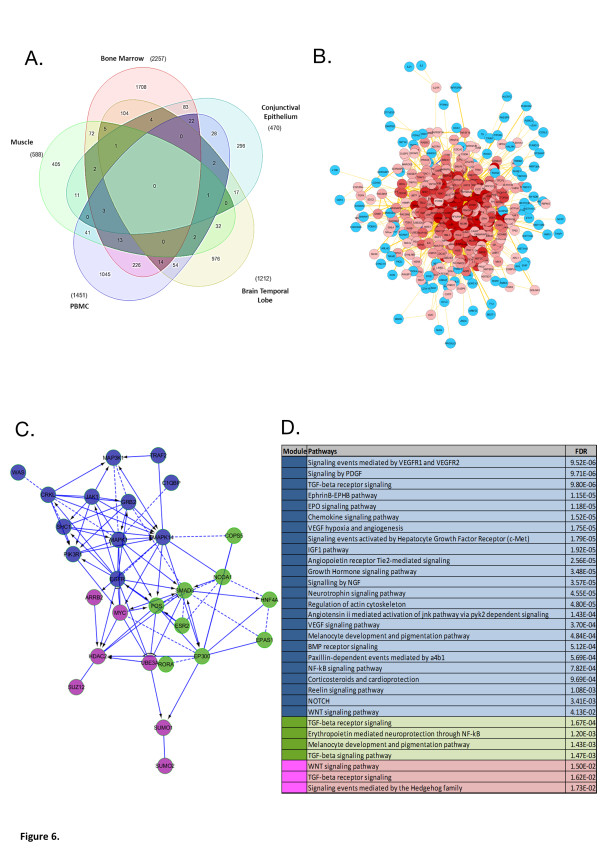
**Analysis of the “overlap” of development-related gene expression between different tissues. A)** Venn Diagram showing overlap of development-related gene expression in five different human types of tissue (peripheral blood mononuclear cells, conjunctival epithelia, temporal lobe brain, quadriceps muscle and bone marrow) [ANOVA, p < 0.05], overlap of three or more data sets shown as darker shade. **B)** Interactome network (Biogrid 3.1.87) model inferred from overlapping genes with development-related expression in ≥3 out of 5 different types of human tissue, protein connectivity (degree) is represented by colour gradation of nodes, dark red = high degree to blue = low degree, as defined by the Cytohubba Cytoscape plugin (426 protein nodes, 447 connections). **C)** Tissue-independent minimal essential network (MEN) derived from genes with development-dependent gene expression. The minimal essential network was constructed using genes within the top 10% score for “degree” and “bottleneck” derived from an interactome network (Biogrid 3.1.87) model inferred from overlapping genes with development-related expression in >3 out of 5 different types of human tissue (peripheral blood mononuclear cells, conjunctival epithelia, temporal lobe brain, quadriceps muscle and bone marrow). A minimal essential network is a marker of functionally relevant biological pathways [15,16], modularity within the MEN was assessed using spectral partition based network clustering [37], the three modules identified are coloured blue, green and pink. Network edges are as follows; “->” for activating/catalyzing, “-|” for inhibition, “- -” for functional interactions extracted from complexes or inputs, and “---” for predicted functional interactions. **D)** Evolutionarily conserved growth pathways identified in the main data set also overlap with the minimal network constructed from the intersection of development-related gene expression in different human tissue. Biological pathways were identified by network module with the Reactome Cytoscape plugin using the Hypergeometric test with false discovery rate modified p-value (FDR).

Examination of other human tissue for development-related changes in gene expression was performed over childhood (>0 to ≤18 years of age) in a variety of available tissue data sets (Additional file 1: Tables S1 & S7, ANOVA, p < 0.05); bone marrow (n = 25, 2257 genes)[32], skeletal muscle (n = 14, 587 genes) [33], PBMCs (n = 53, 1450 genes)[34], conjunctival endothelium (n = 18, 469 genes) [35] and temporal lobe brain tissue (n = 18, 1211 genes) [36]. Infancy and late childhood/puberty clusters of altered gene expression were observed in all tissue comparisons and overlap of gene expression (67 genes, Figure 6A & Additional file 1: Table S7) was used to generate an interactome model of 426 protein nodes and 447 connecting interactions (Figure 6B). A tissue-independent development-related minimal essential network derived from this interactome model (Figure 6C) was associated with the same evolutionarily conserved growth pathways identified in the main data set (Figure 6D, Additional file 1: Table S7). The observations of interactome network connectivity changing with age made in the main lymphoid data set were also seen in brain tissue where adult samples were also available (Additional file 2: Figure S6).

## Discussion

This is the first study to examine the changes in gene expression that accompany aging and development in children. We have shown in normal children using cells of lymphoid origin that the expression of 688 genes is associated with age and subsets of these genes form clusters that correlate with phases of growth – infancy/early childhood, childhood/puberty and final height (Figure 1). These clusters of gene expression were examined in relation to an interactome model and shown to include evolutionarily conserved growth pathways (NOTCH, VEGF, TGFB, WNT) (Figures 3 &4) with high levels of ‘connectivity’ implying essential biological functions [16]. We were able to analyse network connectivity across all age groups in both the main lymphoid data set and in temporal lobe brain tissue; this was highest in infancy and reduced to final height in both data sets (Figure 2 & Additional file 2: Figure S6). It is also of note that a proportion of genes with increased expression in adults also have a similar pattern of expression in infancy (Figure 1); these genes conversely have decreased gene expression over puberty and are enriched for cell cycle related genes (p <2.58 × 10^-3^).

This study had some limitations, primarily related to age matching. It was established that each study used to generate the main data set was evenly distributed over childhood for age and gender; however, it was only possible to integrate adults via the lymphoid cells from bone marrow (GSE11504) although the rest of this data set was homogenous with the other sets used. Using temporal lobe brain tissue as an independent data set, it was also shown that the adult development-related gene expression cluster had similar interactome connectivity in comparison to the adult cluster in the main data set, as well as a similar relation to the infancy and late childhood/puberty clusters. Another potential issue is that there is variation in the timing of entry to puberty [38,39] which is not possible to assess in this study, although we used gender as a co-variate.

There are obvious difficulties in obtaining different tissue samples from healthy children. This issue was reflected in the difficulties encountered in matching the age categories for comparison of development-related gene expression in the five different types of tissue available with appropriate annotation (Additional file 1: Table S1, Figure 6). The tissue-independent development-related gene expression observed in the mouse [6] would imply that we would expect to see more overlap in children than we were able to define; this is likely to be due to imperfect age matching between samples. However, the use of network analysis gives confidence to the observations made using interactome models. This is due to several features of this type of analysis; first biological networks exhibit what are termed “small world” properties [40] implying that fluctuations of connectivity within the network form secondary structure (clusters) associated with function [15,16]; second, as a consequence of the small world feature, biological networks tend to exhibit a “scale free” nature so comparison of networks derived from different sized sets of gene expression data is possible [41,42]; third biological networks are highly robust and resistant to random error [43] therefore tolerating the uncertainty inherent in the comparison of data sets from studies designed for different purposes. All these features of the network analysis of biological systems gives confidence to the observation that an interactome model generated from the 67 genes we identified as “tissue-independent” was associated with the same evolutionarily conserved growth pathways as identified using the main data set despite the small size of the overlap.

We have shown that the expression of clusters of genes varies in a development-dependent manner in multiple human tissues. Whilst the similarity in a development-related gene expression between different tissues was limited (Additional file 1: Table S1, Figure 6A) interactome analysis demonstrated that these changes were integrated into similar evolutionarily conserved growth pathways (Figure 6D). These observations suggest that a “re-wiring” of highly connected interactome modules is a primary difference between tissues, as has been previously suggested by Bossi & Lehner [44] and that growth occurs via similar mechanisms.

Several of the pathways identified vary between infancy and puberty in all tissues examined (TGFB, VEGF, NOTCH, GH & IGF-I) (Figures 3 &6). However both the GR and WNT signalling pathways were clearly associated with all three growth phase-related clusters; this may reflect the known association of these pathways with aspects of the human aging process [45-49]. Also WNT signalling was identified as part of the genetic program for growth observed in the mouse with a temporal change in the three tissues studied (heart, lung and kidney) [6].

The association of glucocorticoid receptor signalling with development-related gene expression was undertaken separately (Figure 5) by using known GR interactions as markers of activity. The GR pathway is modulated by the hypothalamic–pituitary–adrenal axis and has organism wide effects on different tissue. The primary mechanism of glucocorticoid signalling is genomic (i.e. as a transcription factor) and not mediated by cell surface receptors; tissue specificity is modulated by enzymatic conversion of the ligand to an inactive form (cortisol to cortisone), tissue-specific expression of co-regulators and/or epigenetic effects [50,51]. The pleiotropic and organism-wide effects of GR signalling indicate a specific niche in the integration of organismal responses. The observation of glucocorticoid regulation strongly associated with development-related pathways is in alignment with this model of GR signalling, and how multiple tissue and time-dependent events can be regulated by GR [52,53].

We examined whether the development-related genes identified in our study were also present in other investigations into mammalian height growth. Genome-wide association studies by the Genetic Investigation of ANthropometric Traits (GIANT) consortium have defined a set of genes associated with adult height and mapped these genetic differences to growth-related biological pathways including the NOTCH & TGFB signalling pathways, also identified by network analysis in our work [54]. Development-dependent changes have been observed in the methylation of human genes using PBMCs although this work was not correlated with phases of human growth [21]; 12% of the genes with development-related gene expression also have age-associated methylation changes indicating that epigenetic mechanisms are important in the transition of human growth phases. In mouse embryonic fibroblasts the whole genome response to growth hormone has been investigated using STAT5 as a marker [22]. Of the development-related genes defined in our study we identified 18% by orthology that also had been shown to bind STAT5 in the mouse and therefore are potentially growth hormone responsive.

The evolutionary origin of the growth pathways associated with the clusters of development-related gene expression defined in this study dates back to insects in the case of the glucocorticoid receptor pathway [55] and in most cases extends back to the origins of multi-cellularity e.g. WNT, TGFB, VEGF and NOTCH pathways [56] (Figures 3 &6). These data suggest that a similar pathway signature should be present in multiple types of human tissue and that a genetic program exists to regulate the development of the phases of child growth. The further definition of development-related gene expression in longitudinal studies will allow more detailed investigation of this event and provide insight into the return to long bone growth in puberty as a specifically human event [2]; also it provides a framework on which a “human growth program” could exist analogous to that defined in rodents [6].

It is likely that development-related gene expression within some biological pathways has implications for the treatment and pathogenesis of disease. The possible association of age and growth-related gene expression changes with childhood cancers has been highlighted for rhabdomyosarcoma: imprinted growth genes (*Igf2*, *Mest*, *Plagl1*, *Peg3* &*Dlk1*), whose decreased expression is associated with growth deceleration in rodents [6], show increased expression in human rhabdomyosarcoma cell lines [8]. However it is likely that many other genes are involved in human growth which is characterised not only by periods of deceleration (infancy to childhood, as seen in the rodent [2]) but also acceleration (minor in adrenarche, major in puberty, the latter unique to humans [2]). The key evolutionarily conserved growth pathways associated with a development-related gene expression in this study are involved in the aetiology and pathogenesis of childhood cancers e.g. TGFβ signalling in Wilms tumour [57] and Rhabdomyosarcoma [58]. Biomarkers in use from these pathways include FOXO1 within the VEGF pathway, as a prognostic marker in rhabdomyosarcoma (as a fusion gene with PAX3 [59]) and also shown to be highly expressed in the infancy cluster of increased gene expression (Additional file 1: Table S2).

The mathematical properties of biological networks (network topology) have been associated with biological function using protein connectivity as a primary measure [16,60]. Proteins from genes with a development-related gene expression changes have been shown to have significant differences in “connectivity” within the interactome, with greatest “connectivity” shown in infancy and decreasing to final height in adults (Figure 2 & Additional file 2: Figure S6). Using analysis of the interactome we have defined a “map” of normal growth. Comparison of gene expression in childhood diseases/conditions with this map in normal children will allow the characterisation of potential abnormalities in the timing and/or levels of expression in gene networks therefore establishing whether there is a maturational aspect to the disease/condition and hence contribute to understanding mechanism or the identification of targets for diagnosis or prognosis.

## Conclusions

These results imply the existence of a tissue-independent genetic program that correlates with age and phase of human growth. Furthermore our work demonstrates the first observations of gene expression changes within evolutionarily ancient pathways associated with a distinctly human growth event, the pubertal growth spurt, a recent and human-specific addition to mammalian growth. These observations have direct medical relevance as an abnormality in switching the program from one phase to the next, e.g. failure to “turn off” infancy genes, may lead to pathological changes that induce disease. Taking into account development-dependent gene expression profiles for normal children will be the key to the appropriate selection of genes and pathways as potential biomarkers of disease or as drug targets.

## Methods

### Gene expression datasets

The only tissue readily available for expression profiling in children is peripheral blood mononuclear cells. This model has been validated as suitable for analysis of gene expression in a number of childhood conditions [61-63], including both immune mediated disease [64,65] and non-immune disease e.g. Diabetes [61]. Specifically there is precedent from many studies that provide validation of the use of PBMCs to study responses to growth hormone (GH) therapy [9,66-68] thus demonstrating the utility of using lymphoid cells to study human development.

Gene expression analysis was conducted on a library of gene expression datasets from normal children with age annotation collated from the NCBI Gene expression Omnibus (GEO) and EBI Arrayexpress databases (Additional file 1: Table S1.). Age and gender distribution over childhood were shown to overlap between the different data sets used (Additional file 1: Table S1) and the effects of data set variation distribution analysed by multi-dimensional scaling. The original Affymetrix CEL files from GSE9006 [61], GSE26440 [62], GSE11504 [32] and TABM666 [63] were downloaded and combined into one group to form a main analysis data set following published guidelines [69]. All original data were screened for batch effects, different Affymetrix probe-sets were matched (Netaffx [70]) and then the data were combined.

A PBMC data set (GSE 20307) [34] was used to replicate the observation of development-associated differential gene expression in children from the main lymphoid tissue derived data set (Additional file 1: Table S7). The presence of development-dependent gene expression in different tissues was investigated in five different tissues using gene expression data from normal children (GSE11504– bone marrow [32], GSE 6011 – muscle [33], GSE 20307 – PBMCs [34], GSE20436 – Conjunctival epithelium [35] and GSE37721 Temporal lobe brain tissue [36]). Age and gender distribution over childhood were assessed and shown to overlap between the different data sets used (Additional file 1: Table S1).

### Normalisation and quality control of gene expression data

The four candidate datasets (three from PBMCs/whole blood [61-63] and one from bone marrow [32]) were combined to form a main data set following guidelines for the conduct of microarray meta-analyses [69]. The raw CEL files from all data sets were downloaded from GEO and for background correction the Robust Multichip Average (RMA) was applied to the combined data, pre-adjusted for GC content with quantile normalisation and a mean probe set summarisation using Partek® Genomics Suite™ software (version 6.12).

The data set generated was subject to rigorous quality control to investigate the presence of outliers and further confounding effects. Model formulation consisted of three stages; first, dimensional scaling using Principal Components Analysis (PCA) and Iso-map multidimensional scaling (MDS) [71,72] was used to demonstrate data homogeneity (Qlucore Omics Explorer 2.2) and identify outliers using cross-validation (Additional file 2: Figure S1 A&B); secondly, to assess the effects of different distributions of age and gender in each separate data set a sliding window multi-dimensional scaling approach was used. In this method four separate age groups were compared at one time across the entire data range with no observable sub-clustering within the data (examples in Additional file 2: Figure S1 C&D). Finally, overlap of development-related response between the different datasets was confirmed using analysis by ANOVA in each separate dataset (Additional file 2: Figure S1 E-H & Additional file 1: Table S1). The application of these analyses established the suitability for combination of the datasets as independent consistency of overall data along with the absence of confounding effects were demonstrated.

A final set of gene expression probe-sets ready for further analysis was generated using a variance cut-off relative to the variable with the largest variance (σ_max_) to remove non-informative probes, set at 0.1 σ/σ_max_ (Qlucore Omics Explorer 2.2) resulting in a final set of 24839 expression probes in the main data set.

### Statistical analysis of gene expression data

Analysis of variance (ANOVA) with Benjamini–Hochberg correction for false discovery rate was used to determine differential gene expression between groups, with both gender and study used as co-variates in the analysis. Supervised hierarchical clustering was performed on the co-variant normalised data using Kendell’s dissimilarity and Ward’s method on data normalized to a mean of zero and a variance of one (Partek® Genomics Suite™ software version 6.12). The substitution of randomly selected genes was used to assess the specificity of the clusters observed over ten iterations (QlucoreOmics Explorer).

### Gene ontolgy

The identification of enriched gene ontology (GO) of biological pathways was performed within WebGestalt [73] using the Hypergeometric test with a Benjamini-Hochberg correction for multiple testing and confirmed using the BINGO plugin [74] for Cytsoscape 2.8.2 and DAVID Bioinformatics Resources 6.7 [75]. Biological pathway ontology was assessed using the **K**yoto **E**ncyclopedia of **G**enes and **G**enomes database (**Kegg**[76]). Enriched gene functions were also identified using Partek® Genomics Suite™ software and Ingenuity Pathways Analysis (IPA) using Fisher’s exact test. Gene ontology (GO) ANOVA was performed in Partek® Genomics Suite™ software using an enrichment cut-off of q < 0.2. Overlap of gene ontology data was visualised by Venn Diagrams generated using Biovenn [77] and the statistical significance of overlap was assessed using a right-sided Fisher’s exact test.

Gene ontology was also confirmed using functional interaction networks derived from the development-associated gene expression clusters analysed by the Reactome Plugin [27-29] for Cytoscape and the web-based ‘Search Tool for the Retrieval of Interacting Genes/Proteins’ (STRING) [78,79].

### Transcription factor analysis

Upstream regulator analysis of differentially expressed gene clusters was performed using Ingenuity Pathway Analysis software (IPA). This method is based on expected causal effects between upstream regulators and targets; the expected causal effects are derived from the literature compiled in the Ingenuity Knowledge Base. A prediction of the activation state for each transcription factor based on the direction of change was calculated (z-score) using the gene expression patterns of the transcription factor and its downstream genes. An absolute z-score of ≥ |2| was considered significant. A p-value was also calculated by Fisher’s Exact Test indicating the statistical significance of genes in the dataset that are downstream of the transcription factor.

### Network analysis

Network analysis was performed to increase confidence in the observations of differentially expressed genes by correlation with biological pathways. This process also allowed the identification of putative key functional elements within the networks of differentially expressed genes.

Three different software based methods were used to generate and analyse biological networks, all of which used algorithms to infer the relationship of differentially expressed genes with known interactions in the literature or from databases. The Biogrid model of the human Interactome (31.1.87) was used to generate networks associated with development-related genes. Results from these networks were confirmed and pathway ontology was added using Ingenuity Pathway Analysis Software and the Reactome FI (Functional Interaction) plugin for Cytoscape.

1 Biogrid 2.0 Cytoscape 2.6.0 Plugin [24]. A filter was created for the Biogrid human interactome model (31.1.87) using the development-related gene clusters. The resultant networks were visualised in Cytoscape.

2 Ingenuity Pathways Analysis (IPA) software. Differentially expressed genes serve as “seeds” for generating networks and are combined into networks that maximize their connectivity in relation to specific biological functions. Networks are scored based on the number of Network Eligible Molecules in the network and its size, as well as the total number of Network Eligible Molecules analysed and the total number of molecules in the Ingenuity Knowledge Base that could potentially be included in networks [6]. The score is the –log of the Fisher’s Exact test result. The score is not an indication of the quality or biological relevance of the network; it calculates the approximate “fit” between each network and the differentially expressed genes [80].

3 Reactome FI Cytoscape 2.8.2 Plugin [81,82]. This plugin accesses the Reactome Functional Interaction (FI) network [27,29], a manually curated, peer-reviewed, pathway-based protein functional interaction network covering close to 50% of human proteins, and allows the construction of FI networks based on a set of genes. The FI network is clustered to form highly-interacting groups of genes (spectral partition based network clustering) [37], perform gene ontology (GO) functional enrichment analysis to annotate the modules and expand the network by finding genes related to the experimental data set [27,29,83].

### Network properties and minimal essential networks

Network properties have been correlated with biological function [16]. The Cytohubba Cytoscape Plugin was used to provide topological analysis and allowed the definition of a range of network properties including Degree (“Hubness”) and Bottleneck (BN). The top 10% of PPI network nodes ranked for both “degree” and “Bottleneck” scores were used to evaluate node “essentiality” [15,84] and to generate a minimal essential network (MEN) (Additional file 2: Figure S5). A minimal essential network represents the most functionally relevant element of an interactome model and therefore was used to assess biological function [15,16,66].

### Availability of supporting data

The data sets supporting the results of this article are available in the Gene Expression Omnibus repository:

GSE9006 (http://www.ncbi.nlm.nih.gov/geo/query/acc.cgi?acc=GSE9006),

GSE26440 (http://www.ncbi.nlm.nih.gov/geo/query/acc.cgi?acc=GSE26440),

GSE11504 (http://www.ncbi.nlm.nih.gov/geo/query/acc.cgi?acc=GSE11504),

TABM666 (http://www.ebi.ac.uk/arrayexpress/experiments/E-TABM-666),

GSE6011 (http://www.ncbi.nlm.nih.gov/geo/query/acc.cgi?acc=GSE6011),

GSE37721 (http://www.ncbi.nlm.nih.gov/geo/query/acc.cgi?acc=GSE37721),

GSE20307 (http://www.ncbi.nlm.nih.gov/geo/query/acc.cgi?acc=GSE20307),

GSE20436 (http://www.ncbi.nlm.nih.gov/geo/query/acc.cgi?acc=GSE20436).

## Competing interests

B.D. was an employee of Merck Serono S.A., Geneva, Switzerland.

## Authors’ contributions

AS conceived the idea for evaluating development-related gene expression in normal children as a background to evaluating gene expression changes in growth disorders. AS performed the micro-array and network analyses and developed the minimal essential network as a study tool. DH contributed to and supported network analysis of data, with AW and BD supporting micro-array normalisation, quality control and analysis. AS, PCh & PC designed the analysis plan and the presentation of the data. A.S and P.C. led the writing of the manuscript with input from all authors. All authors read and approved the final manuscript.

## Supplementary Material

Additional file 1: Table S1 Library of gene expression datasets from normal children with age annotation collated from the NCBI Gene expression Omnibus (GEO) and EBI Arrayexpress databases. **Table S2**: Genes with age related expression in main data set. 927 Affymetrix probesets identified by ANOVA (q < 0.1). Three groups defined by hierarchical clustering, ≤6 years of age [infancy, early childhood group (408 probes)]; >6 to ≤17 years of age [late childhood, puberty group (252 probes)]; and >17 to <30 years of age [adulthood (267 probes)]. Ranked by q value. **Table S3**: Enriched KEGG pathways associated with age-related gene expression controlled against the background of the human genome. **Table S4**: Genes previously identified in genome wide association studies (GWAS) that also have age-related expression in humans. GWAS data from the Catalogue of Published Genome-Wide Association Studies at the National Human Genome Institute (Hindorff *et al.* 2009). **Table S5**: Gene expression associated essential biological pathways defined by network analysis. **Table S6**: Replication of gene expression associated essential biological pathways defined by network analysis in peripheral blood mononuclear cell (PBMC) gene expression data. **Table S7**: Overlap of gene expression associated essential biological pathways defined by network analysis between different human tissues. Age-related gene expression was identified in five different human tissues, bone marrow, quadriceps muscle, peripheral blood mononuclear cells, temporal lobe brain and conjunctival epithelia (ANOVA, p < 0.05). Overlap of these gene sets was examined (Additional file 2: Figure S8) and a set of genes where age related expression occurred in at least three of the five tissues was identified (n = 68). Click here for file

Additional file 2: Figure S1 Generation of the main data set. **Figure S2**: Age related differences in gene ontology. **Figure S3**: Age related differences in expression of genes within canonical pathways. Biological pathways were associated with the three clusters of age related genes as identified from the KEGG database (Webgestalt); ≤6 yrs [Infancy, Early Childhood]; >6 to ≤17 yrs [Late Childhood, Puberty] and >17 yrs [Adult, Final Height] (hypergeometric test, q < 0.2). **Figure S4**: Identification of transcription factors that are expected to be activated or inhibited, given the observed gene expression changes in the three clusters of age related genes; ≤6 yrs [Infancy, Early Childhood]; >6 to ≤17 yrs [Late Childhood, Puberty] and >17 yrs [Adult, Final Height]. **Figure S5**: Analysis of network topology. **Figure S6**: Analysis of protein connectivity (degree) in the human interactome as a measure of protein function within genes within age-related expression clusters from temporal lobe human brain tissue (GSE37721, Sterner *et al.* 2012). Click here for file
