# Supplementary material for: Human growth is associated with distinct patterns of gene expression in evolutionarily conserved networks
Source: BMC Genomics. 2013 Aug 13;14:547. doi: 10.1186/1471-2164-14-547 (PMC3765282; doi:10.1186/1471-2164-14-547)
Supplement: Additional file 2: Figure S1 — Generation of the main data set. Figure S2: Age related differences in gene ontology. Figure S3: Age related differences in expression of genes within canonical pathways. Biological pathways were associated with the three clusters of age related genes as identified from the KEGG database (Webgestalt); ≤6 yrs [Infancy, Early Childhood]; >6 to ≤17 yrs [Late Childhood, Puberty] and >17 yrs [Adult, Final Height] (hypergeometric test, q < 0.2). Figure S4: Identification of transcription factors that are expected to be activated or inhibited, given the observed gene expression changes in the three clusters of age related genes; ≤6 yrs [Infancy, Early Childhood]; >6 to ≤17 yrs [Late Childhood, Puberty] and >17 yrs [Adult, Final Height]. Figure S5: Analysis of network topology. Figure S6: Analysis of protein connectivity (degree) in the human interactome as a measure of protein function within genes within age-related expression clusters from temporal lobe human brain tissue (GSE37721, Sterner et al. 2012). [file 1471-2164-14-547-S2.pptx]

## Slide 1
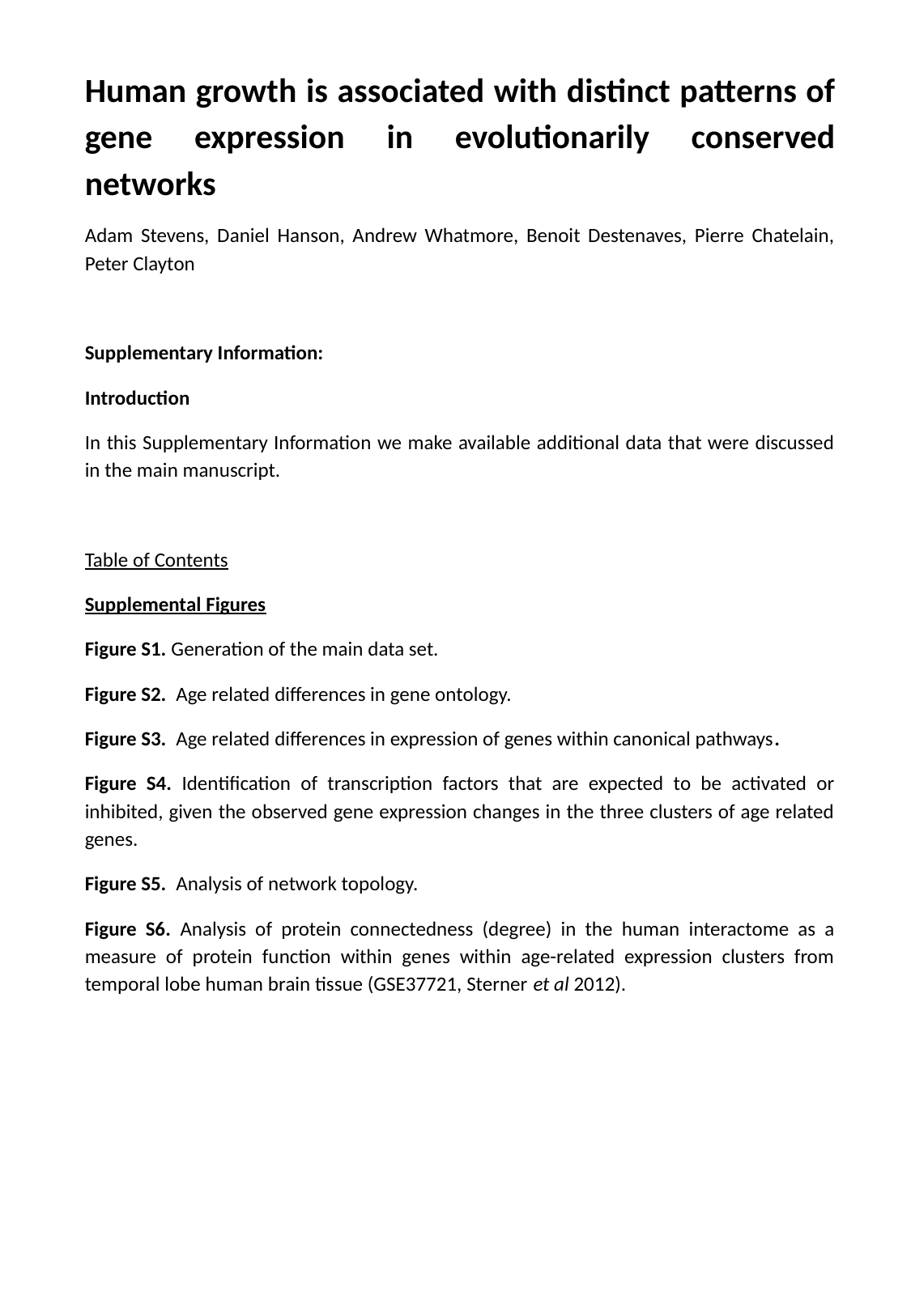

Human growth is associated with distinct patterns of gene expression in evolutionarily conserved networks
Adam Stevens, Daniel Hanson, Andrew Whatmore, Benoit Destenaves, Pierre Chatelain, Peter Clayton
Supplementary Information:
Introduction
In this Supplementary Information we make available additional data that were discussed in the main manuscript.
Table of Contents
Supplemental Figures
Figure S1. Generation of the main data set.
Figure S2. Age related differences in gene ontology.
Figure S3. Age related differences in expression of genes within canonical pathways.
Figure S4. Identification of transcription factors that are expected to be activated or inhibited, given the observed gene expression changes in the three clusters of age related genes.
Figure S5. Analysis of network topology.
Figure S6. Analysis of protein connectedness (degree) in the human interactome as a measure of protein function within genes within age-related expression clusters from temporal lobe human brain tissue (GSE37721, Sterner et al 2012).

## Slide 2
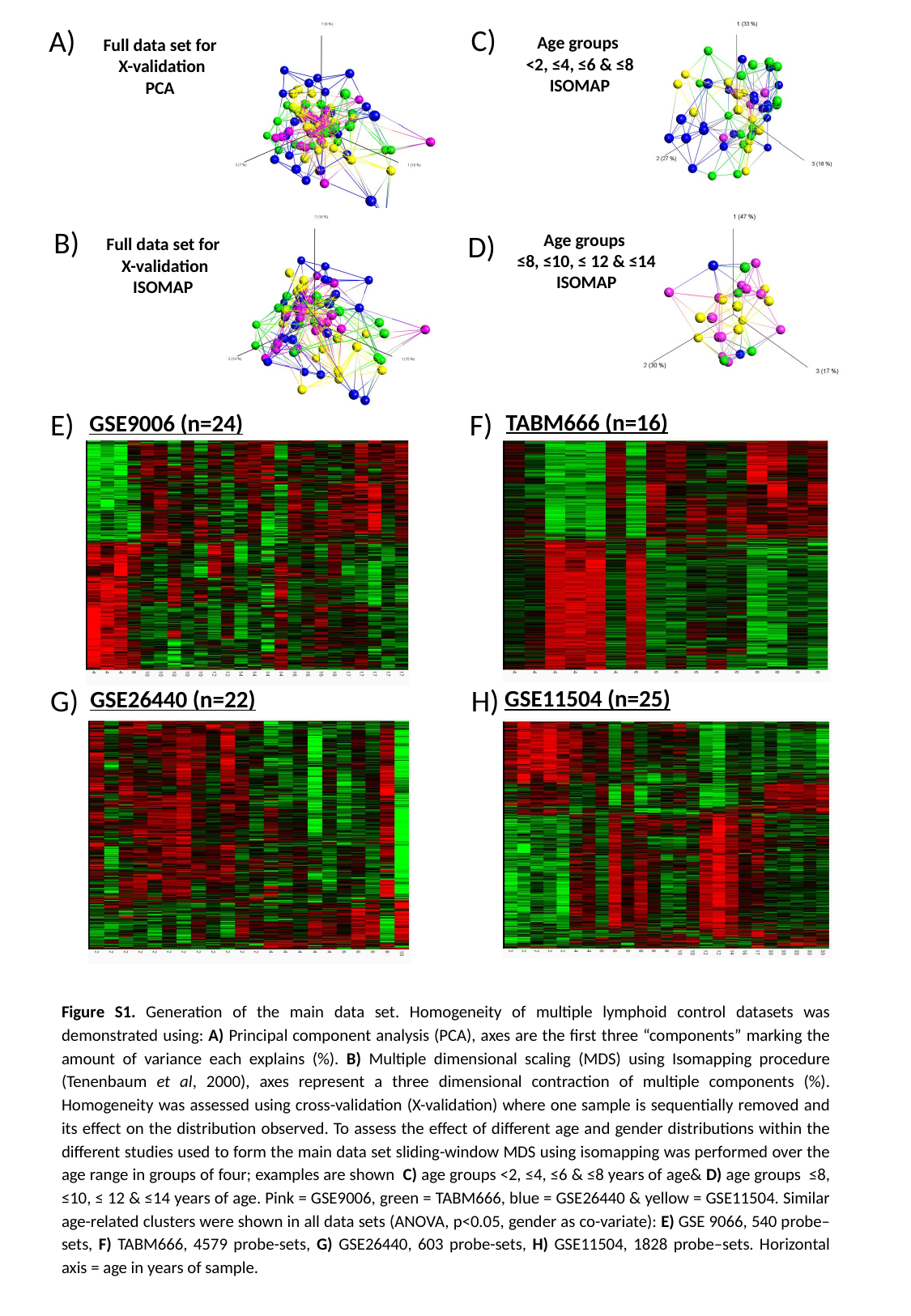

C)
A)
Age groups
<2, ≤4, ≤6 & ≤8
ISOMAP
Full data set for
 X-validation
PCA
B)
D)
Age groups
≤8, ≤10, ≤ 12 & ≤14
ISOMAP
Full data set for
 X-validation
ISOMAP
E)
F)
TABM666 (n=16)
GSE9006 (n=24)
G)
H)
GSE11504 (n=25)
GSE26440 (n=22)
Figure S1. Generation of the main data set. Homogeneity of multiple lymphoid control datasets was demonstrated using: A) Principal component analysis (PCA), axes are the first three “components” marking the amount of variance each explains (%). B) Multiple dimensional scaling (MDS) using Isomapping procedure (Tenenbaum et al, 2000), axes represent a three dimensional contraction of multiple components (%). Homogeneity was assessed using cross-validation (X-validation) where one sample is sequentially removed and its effect on the distribution observed. To assess the effect of different age and gender distributions within the different studies used to form the main data set sliding-window MDS using isomapping was performed over the age range in groups of four; examples are shown C) age groups <2, ≤4, ≤6 & ≤8 years of age& D) age groups ≤8, ≤10, ≤ 12 & ≤14 years of age. Pink = GSE9006, green = TABM666, blue = GSE26440 & yellow = GSE11504. Similar age-related clusters were shown in all data sets (ANOVA, p<0.05, gender as co-variate): E) GSE 9066, 540 probe–sets, F) TABM666, 4579 probe-sets, G) GSE26440, 603 probe-sets, H) GSE11504, 1828 probe–sets. Horizontal axis = age in years of sample.

## Slide 3
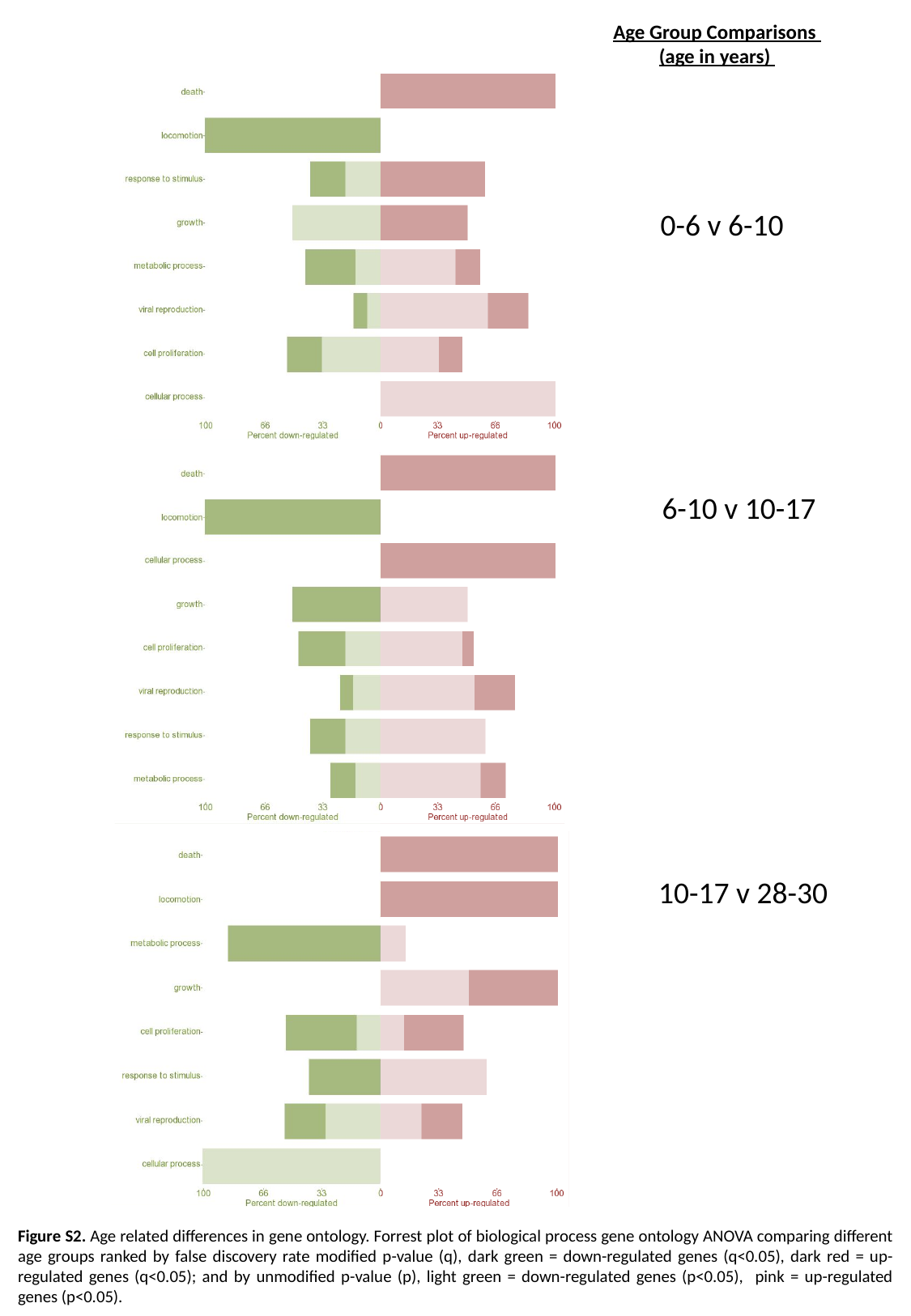

Age Group Comparisons
(age in years)
0-6 v 6-10
6-10 v 10-17
10-17 v 28-30
Figure S2. Age related differences in gene ontology. Forrest plot of biological process gene ontology ANOVA comparing different age groups ranked by false discovery rate modified p-value (q), dark green = down-regulated genes (q<0.05), dark red = up-regulated genes (q<0.05); and by unmodified p-value (p), light green = down-regulated genes (p<0.05), pink = up-regulated genes (p<0.05).

## Slide 4
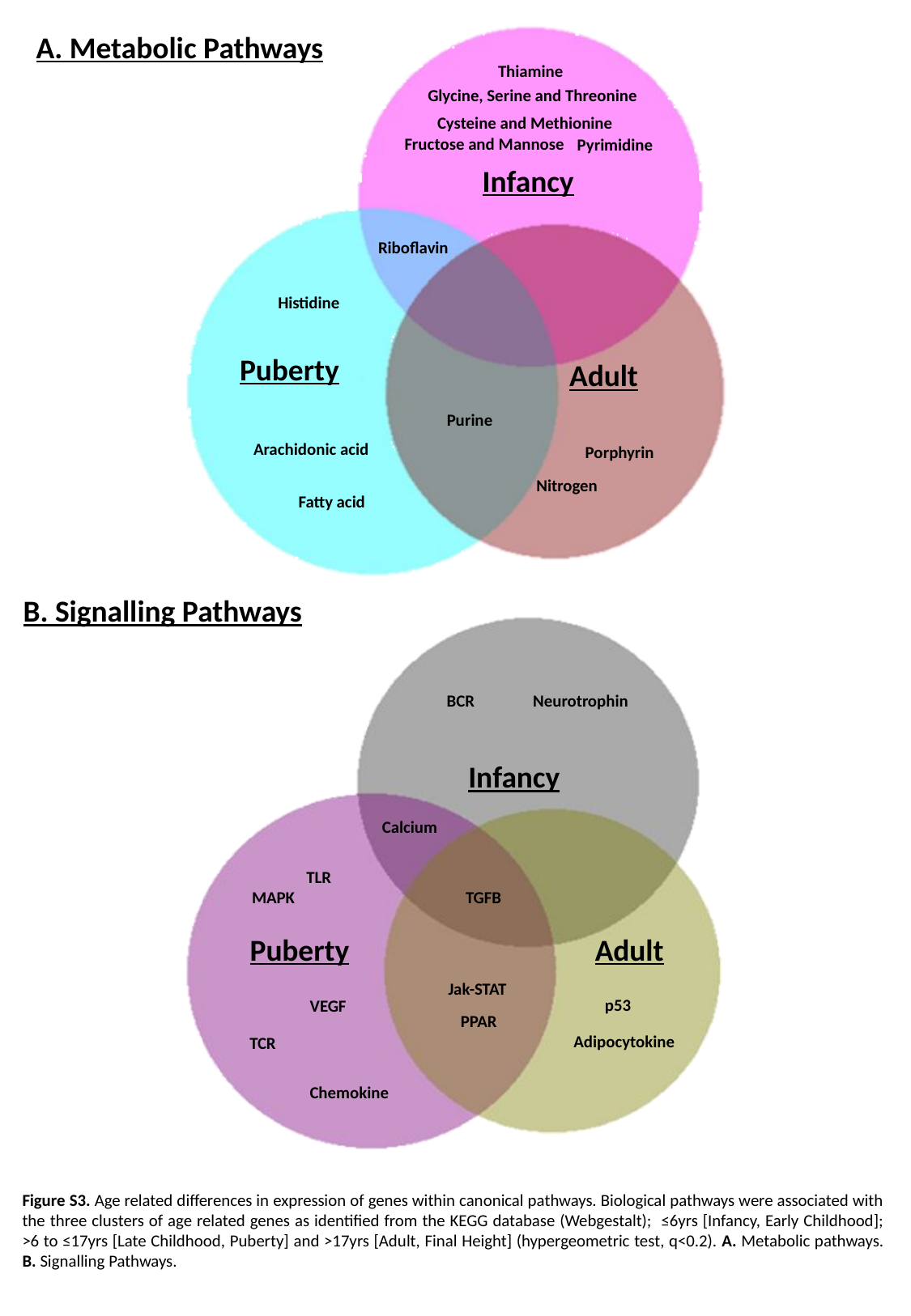

A. Metabolic Pathways
Thiamine
Glycine, Serine and Threonine
Cysteine and Methionine
Fructose and Mannose
Pyrimidine
Infancy
Riboflavin
Histidine
Puberty
Adult
Purine
Arachidonic acid
Porphyrin
Nitrogen
Fatty acid
B. Signalling Pathways
BCR
Neurotrophin
Infancy
Calcium
TLR
MAPK
TGFB
Puberty
Adult
Jak-STAT
p53
VEGF
PPAR
Adipocytokine
TCR
Chemokine
Figure S3. Age related differences in expression of genes within canonical pathways. Biological pathways were associated with the three clusters of age related genes as identified from the KEGG database (Webgestalt); ≤6yrs [Infancy, Early Childhood]; >6 to ≤17yrs [Late Childhood, Puberty] and >17yrs [Adult, Final Height] (hypergeometric test, q<0.2). A. Metabolic pathways. B. Signalling Pathways.

## Slide 5
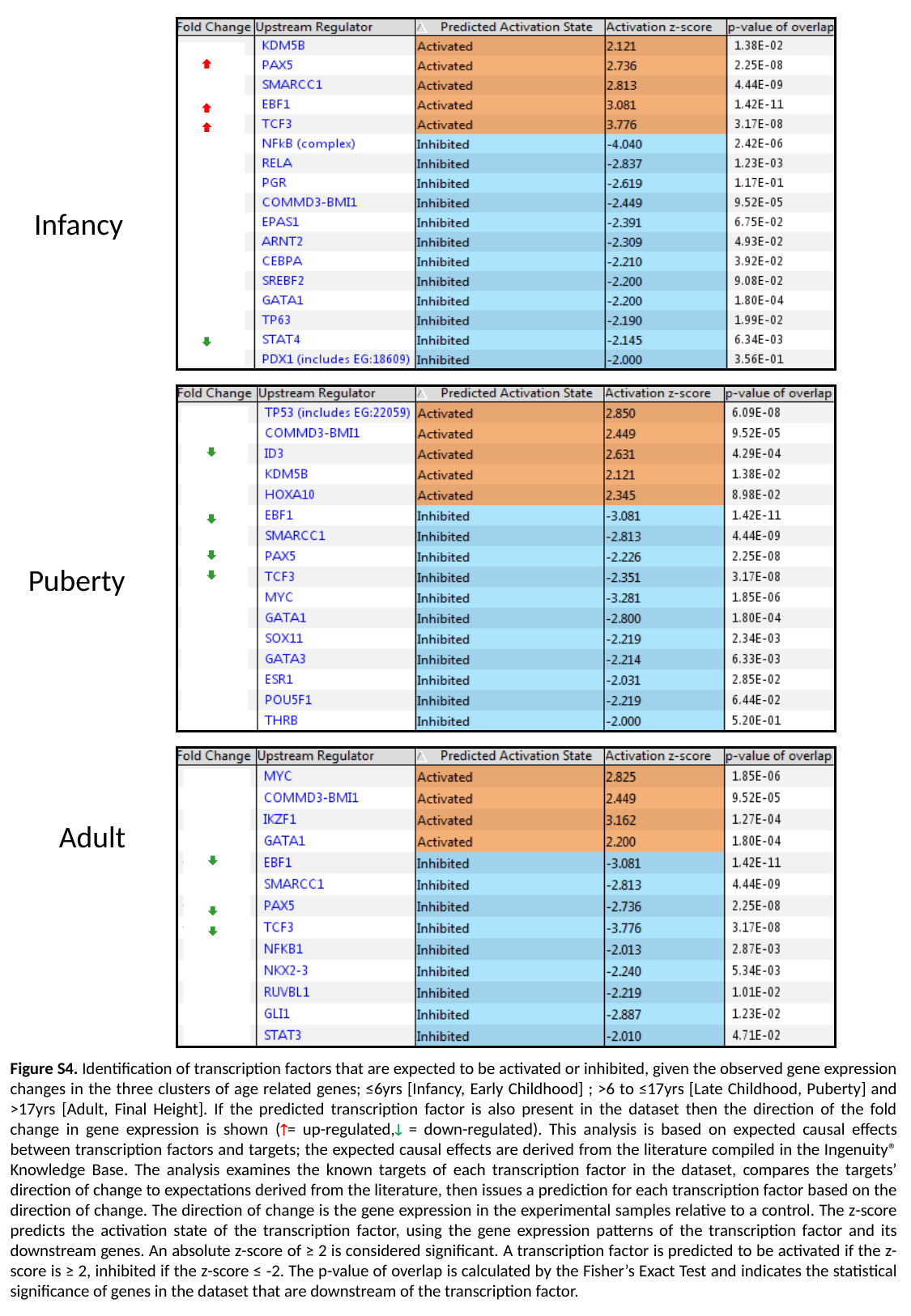

Infancy
Puberty
Adult
Figure S4. Identification of transcription factors that are expected to be activated or inhibited, given the observed gene expression changes in the three clusters of age related genes; ≤6yrs [Infancy, Early Childhood] ; >6 to ≤17yrs [Late Childhood, Puberty] and >17yrs [Adult, Final Height]. If the predicted transcription factor is also present in the dataset then the direction of the fold change in gene expression is shown (= up-regulated, = down-regulated). This analysis is based on expected causal effects between transcription factors and targets; the expected causal effects are derived from the literature compiled in the Ingenuity® Knowledge Base. The analysis examines the known targets of each transcription factor in the dataset, compares the targets’ direction of change to expectations derived from the literature, then issues a prediction for each transcription factor based on the direction of change. The direction of change is the gene expression in the experimental samples relative to a control. The z-score predicts the activation state of the transcription factor, using the gene expression patterns of the transcription factor and its downstream genes. An absolute z-score of ≥ 2 is considered significant. A transcription factor is predicted to be activated if the z-score is ≥ 2, inhibited if the z-score ≤ ‑2. The p-value of overlap is calculated by the Fisher’s Exact Test and indicates the statistical significance of genes in the dataset that are downstream of the transcription factor.

## Slide 6
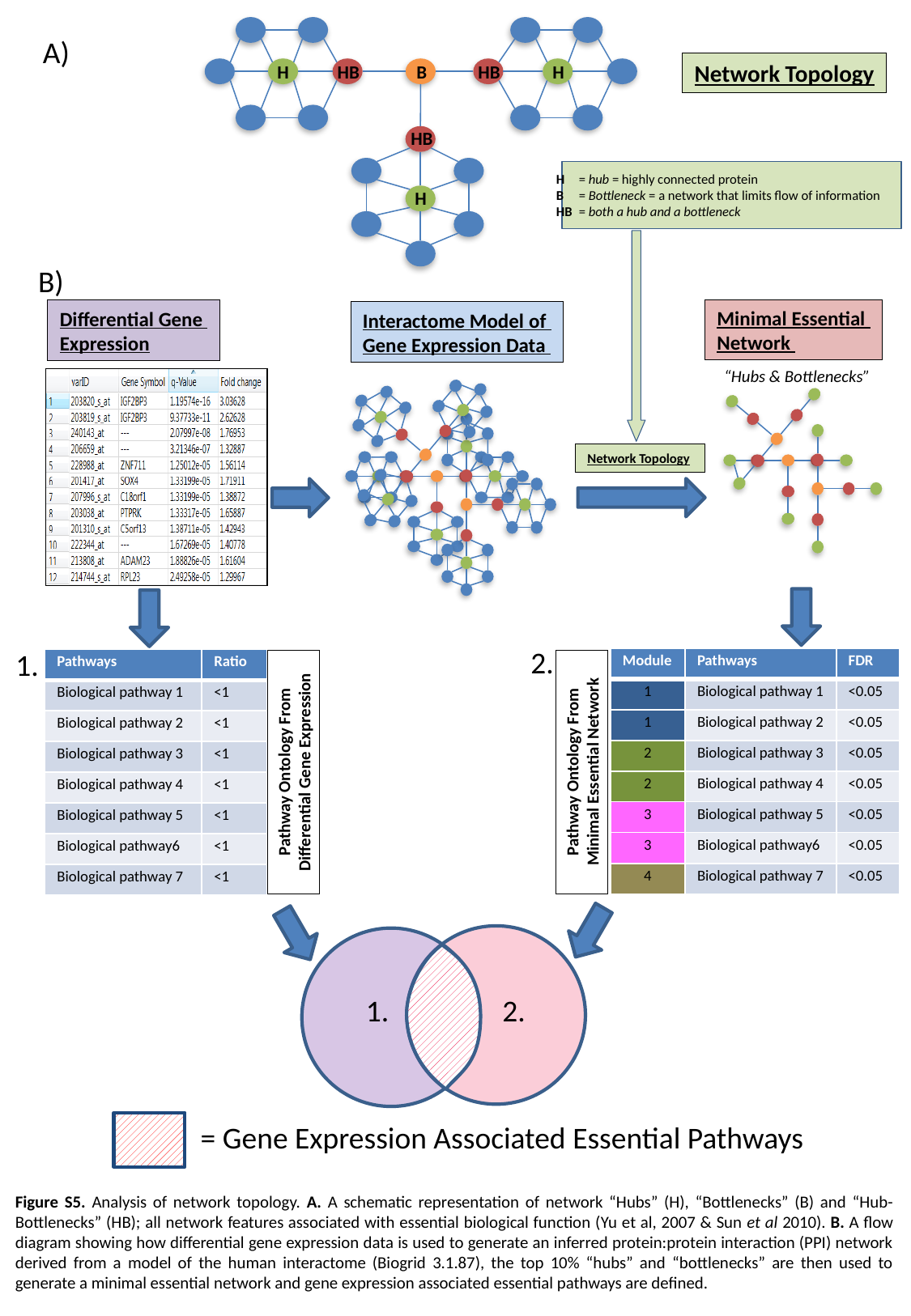

HB
H
HB
B
H
HB
H
A)
Network Topology
H	 = hub = highly connected protein
B	 = Bottleneck = a network that limits flow of information
HB 	 = both a hub and a bottleneck
B)
Minimal Essential
Network
Differential Gene
Expression
Interactome Model of
Gene Expression Data
“Hubs & Bottlenecks”
Network Topology
2.
1.
| Module | Pathways | FDR |
| --- | --- | --- |
| 1 | Biological pathway 1 | <0.05 |
| 1 | Biological pathway 2 | <0.05 |
| 2 | Biological pathway 3 | <0.05 |
| 2 | Biological pathway 4 | <0.05 |
| 3 | Biological pathway 5 | <0.05 |
| 3 | Biological pathway6 | <0.05 |
| 4 | Biological pathway 7 | <0.05 |
| Pathways | Ratio |
| --- | --- |
| Biological pathway 1 | <1 |
| Biological pathway 2 | <1 |
| Biological pathway 3 | <1 |
| Biological pathway 4 | <1 |
| Biological pathway 5 | <1 |
| Biological pathway6 | <1 |
| Biological pathway 7 | <1 |
Pathway Ontology From Minimal Essential Network
Pathway Ontology From Differential Gene Expression
2.
1.
= Gene Expression Associated Essential Pathways
Figure S5. Analysis of network topology. A. A schematic representation of network “Hubs” (H), “Bottlenecks” (B) and “Hub-Bottlenecks” (HB); all network features associated with essential biological function (Yu et al, 2007 & Sun et al 2010). B. A flow diagram showing how differential gene expression data is used to generate an inferred protein:protein interaction (PPI) network derived from a model of the human interactome (Biogrid 3.1.87), the top 10% “hubs” and “bottlenecks” are then used to generate a minimal essential network and gene expression associated essential pathways are defined.

## Slide 7
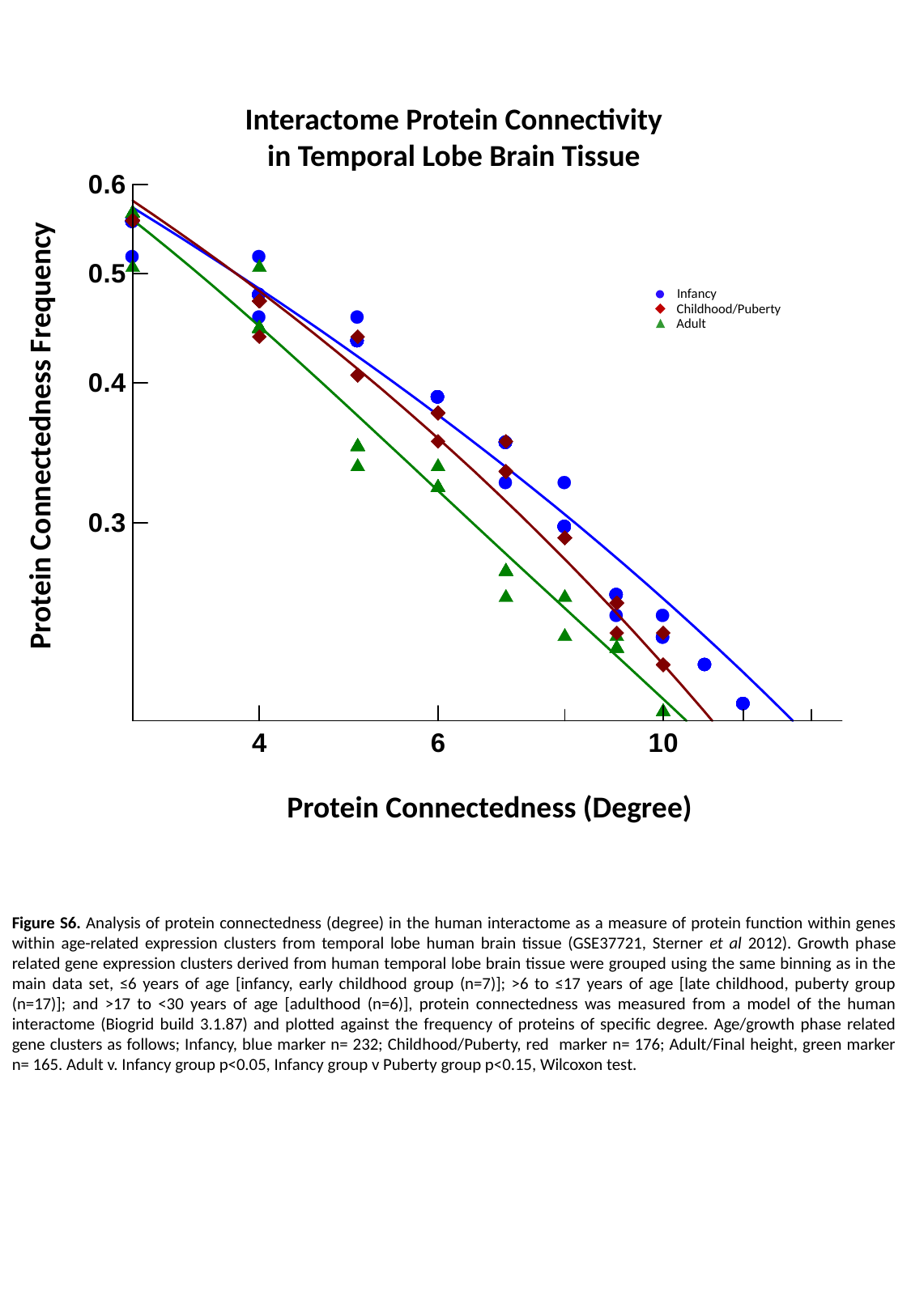

Interactome Protein Connectivity
 in Temporal Lobe Brain Tissue
Infancy
Childhood/Puberty
Adult
Protein Connectedness Frequency
Protein Connectedness (Degree)
Figure S6. Analysis of protein connectedness (degree) in the human interactome as a measure of protein function within genes within age-related expression clusters from temporal lobe human brain tissue (GSE37721, Sterner et al 2012). Growth phase related gene expression clusters derived from human temporal lobe brain tissue were grouped using the same binning as in the main data set, ≤6 years of age [infancy, early childhood group (n=7)]; >6 to ≤17 years of age [late childhood, puberty group (n=17)]; and >17 to <30 years of age [adulthood (n=6)], protein connectedness was measured from a model of the human interactome (Biogrid build 3.1.87) and plotted against the frequency of proteins of specific degree. Age/growth phase related gene clusters as follows; Infancy, blue marker n= 232; Childhood/Puberty, red marker n= 176; Adult/Final height, green marker n= 165. Adult v. Infancy group p<0.05, Infancy group v Puberty group p<0.15, Wilcoxon test.
